# Identification of Adjustment Variables in Indirect Comparisons: A Rapid Review of CAR-T Therapies for Diffuse Large B-Cell Lymphoma

**DOI:** 10.3390/cancers17081335

**Published:** 2025-04-15

**Authors:** Sybille Riou, Stefanie Rungaldier, Jörg Mahlich

**Affiliations:** 1 Miltenyi Biomedicine, Friedrich-Ebert-Straße 68, 51429 Bergisch Gladbach, Germany; 2 Düsseldorf Institute for Competition Economics (DICE), Heinrich-Heine-University Düsseldorf, Universitätsstraße 1, 40225 Düsseldorf, Germany

**Keywords:** indirect comparison, confounder analysis, CAR-T therapies, diffuse large B-cell lymphoma, real world evidence, rapid literature review, methodology

## Abstract

**Simple Summary:**

This study looks at how different factors, known as “confounders”, are accounted for when comparing CAR-T therapies for diffuse large B-cell lymphoma (DLBCL). While CAR-T therapies are approved for treatment, there have not been direct comparisons between them, so indirect methods are used. However, these methods can be influenced by biases, so it is important to adjust for the right factors. The review found 21 articles, but only 11 were suitable for analysis. It showed that different studies used different factors to adjust for confounding, and the methods for choosing these factors were often unclear. To improve the accuracy of comparisons in future studies, there is a need for more standardized ways to select and adjust for confounders.

**Abstract:**

**Background**: Chimeric antigen receptor T-cell (CAR-T) therapies have been approved by the U.S. Food and Drug Administration (FDA) and the European Medicines Agency (EMA) for the treatment of diffuse large B-cell lymphoma (DLBCL), primarily based on single-arm trials or indirect comparisons with stem cell transplantation. However, no direct head-to-head comparisons of CAR-T therapies have been conducted, largely due to their high cost. To assess their true value, indirect treatment comparisons (ITCs) are essential. These comparisons, however, are prone to confounding biases, which necessitate careful adjustments through the identification and measurement of relevant variables. **Materials and Methods**: This study aims to identify the variables used for adjustment in ITCs of CAR-T therapies for DLBCL and examine the methodologies employed to select them. A rapid literature review was conducted in PubMed in September 2023, focusing on ITCs involving CAR-T therapies for DLBCL. The search was based on keywords categorized into three groups: techniques (ITCs and related terms), drugs (CAR-T therapies), and indication (DLBCL). **Results**: The rapid literature review identified 21 articles, of which 11 were selected for analysis. Exclusions were made for articles that did not identify confounders, were letters to editors, or addressed conditions other than DLBCL. Among the 11 selected publications, 10 did not clearly specify the methodology used to identify adjustment variables. A total of 25 potential confounders were identified across the studies, with substantial variability in the set of variables used, reflecting a lack of standardization in confounder selection. Commonly identified confounders included the number of prior treatment lines and Eastern Cooperative Oncology Group Performance Status (ECOG PS), although their inclusion as adjustment variables in ITCs was inconsistent, often due to missing data. **Conclusions**: While the identified confounders are clinically relevant, the methodologies for selecting them remain unclear, resulting in significant variability across studies. Additionally, key variables commonly considered in health technology assessments (HTAs), such as age, sex, and disease severity, were inconsistently incorporated into ITCs. To improve the reliability and consistency of ITC outcomes, there is a pressing need for standardized methodologies for identifying and adjusting for confounders.

## 1. Introduction

Chimeric antigen receptor T-cell (CAR-T) therapy is an innovative form of adoptive cell therapy that involves the collection of a patient’s immune cells through apheresis, followed by ex vivo genetic modification and subsequent reinfusion [1,2]. The first commercially available CAR-T therapies, tisagenlecleucel (Kymriah^®^) and axicabtagene ciloleucel (Yescarta^®^), were approved by the U.S. Food and Drug Administration (FDA) in 2017 and the European Medicines Agency (EMA) in 2018 for the treatment of diffuse large B-cell lymphoma (DLBCL) in patients who have relapsed or failed to respond to two or more prior treatment lines. DLBCL, the most common subtype of non-Hodgkin lymphoma (NHL), accounts for approximately 32.5% of all NHL cases diagnosed annually [3]. In 2021 and 2022, the FDA and the EMA, respectively, granted approval for lisocabtagene maraleucel (Breyanzi^®^) for the same indication. Additionally, in 2022, both agencies expanded the approval of axicabtagene ciloleucel to include second-line treatment for patients who failed frontline rituximab-based chemoimmunotherapy. Lisocabtagene maraleucel received FDA approval for this line of treatment in 2022 and EMA approval in 2023, following head-to-head trials that highlighted the superior benefits of CAR-T therapies compared to high-dose chemotherapy followed by autologous stem cell transplantation (ASCT) [4,5]. Despite these advances, there is a lack of randomized controlled trials (RCTs) comparing the effectiveness of different CAR-T therapies. One major barrier is the prohibitive cost of CAR-T treatments, which in the U.S. can exceed USD 350,000, with additional expenses of up to USD 160,000 for hospital administration and management of adverse events [6,7]. As a result, indirect treatment comparisons (ITCs) have become essential for evaluating the relative value of these therapies. However, ITCs are susceptible to confounding bias, where certain variables may influence both the treatment and the outcome of interest [8]. In particular, unanchored ITCs (based on single-arm trials) require the identification of both prognostic factors and treatment effect modifiers—patient characteristics that are linked to treatment response [9]. In anchored ITCs, on the other hand, adjustments relate to effect modifiers only [10,11].

Identifying both prognostic factors and effect modifiers typically requires a thorough literature review, followed by validation from a panel of clinical experts [12], a process recommended by health technology assessment agencies [13,14]. Building on this approach, we conducted a rapid review of published indirect treatment comparisons (ITCs) involving CAR-T therapies to identify the confounding variables used for adjustment. Our analysis focused specifically on DLBCL, a disease characterized by increasing incidence and a substantial humanistic and economic burden [15,16]. To the best of our knowledge, no review of this nature has been conducted previously.

## 2. Methods

This rapid literature review aimed to identify the variables used for adjustments in indirect comparisons involving CAR-T therapies for DLBCL. Additionally, we sought to examine the methodologies employed by authors in selecting these variables. To achieve this, we conducted an initial rapid review to identify synonyms and related terms relevant to our research question. For example, indirect comparisons may be associated with terms such as MAIC (Matching Adjusted Indirect Comparison), NMA (Network Meta-Analysis), or STC (Simulated Treatment Comparison), while a CAR-T therapy like Yescarta may also be referred to by its generic name, axi-cel, or its therapeutic class.

Based on this, we defined the keywords for our analysis, which were divided into three categories: technique, drug, and indication (Table 1). The literature search was conducted in PubMed in September 2023, with an “OR” operator applied within each section and an “AND” operator used between sections. The search process followed the PRISMA guidelines [17]. No publication date restrictions were applied due to the recent approval of CAR-T therapies (with the first regulatory approval in 2018). Only full-text publications were included in the analysis, along with Supplementary Materials when available. No language restrictions were applied, except for the use of English keywords, and no limitations were placed on the type of publication. Data collection was performed independently by two reviewers, and in cases of discrepancies between reviewers, the third author was consulted to reach a consensus.

The PubMed search strategy was as follows:

(“indirect comparison*” OR “treatment* comparison*” OR “simulated treatment comparison*” OR STC OR “network meta analys*” OR NMA OR “adjusted comparison*” OR “matching adjusted indirect comparison*” OR “comparing efficacy” OR “real world comparison*” OR “comparative efficacy”) AND (“chimeric antigen receptor T-cell therap*” OR “CAR T*” OR tisagenlecleucel OR Kymriah OR “Tisa-cel” OR “axicabtagene ciloleucel” OR “axi-cel” OR Yescarta OR “lisocabtagene maraleucel” OR “liso-cel” OR Breyanzi) AND (“diffuse large B-cell lymphoma” OR “large B-cell lymphoma” OR DLBCL OR LBCL).

## 3. Results

**Figure 1:**
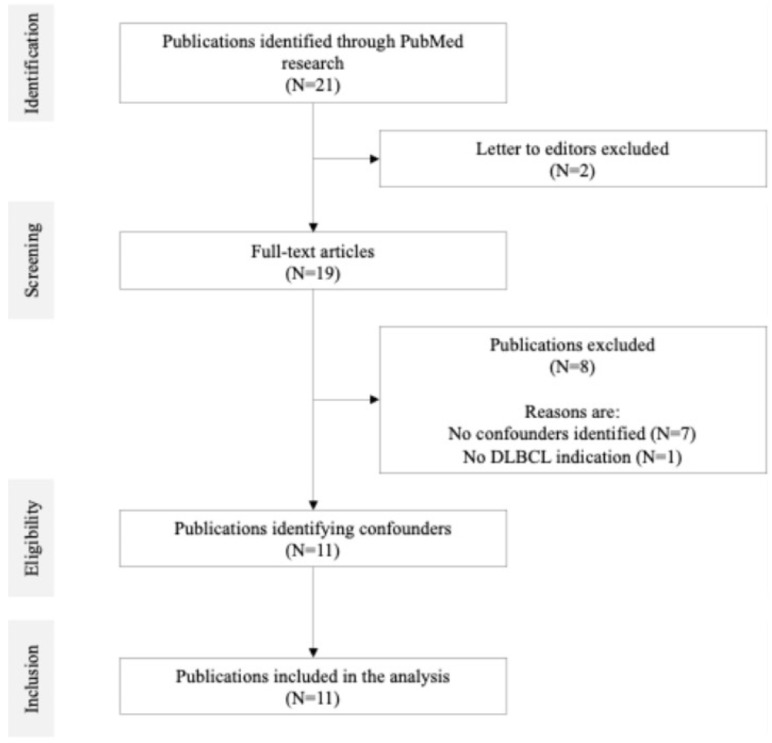
Selection of publications.

Based on our rapid literature review, we identified twenty-one publications. Ten were excluded for the following reasons: confounders were not identified, the articles were letters to the editor related to publications already included, or the indication was not DLBCL (Figure 1).

The eleven publications are summarized in Table 2. All comparisons were based on single-arm trials and were unanchored. Ten of the eleven publications did not specify the methodology used for identifying confounders, instead generally referring to terms such as “literature review” and/or “expert interviews”. One exception employed univariate prognostic analysis, which identifies individual factors influencing treatment outcomes. Various statistical tests can be used for univariate analysis, including the Wilcoxon signed-rank test, logistic regression, t-test, ANOVA, or Mann–Whitney U test. In contrast, multivariate analysis identifies a group of factors affecting treatment outcomes through methods like cluster analysis or latent Dirichlet allocation (LDA), among others [18,19].

**Figure 2:**
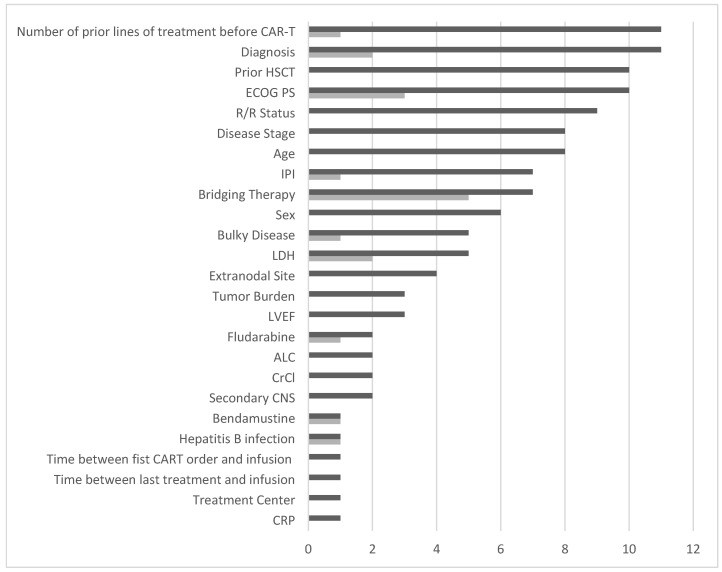
Times a confounder was identified in the publications (dark grey) and times a confounder was identified but not used in the matching and/or sensitivity analysis (light grey). Legend: ALC = absolute lymphocyte count; CrCl = creatinine clearance; CRP = C-reactive protein; ECOG PS = Eastern Cooperative Oncology Group Performance status; HSCT = hematopoietic stem-cell transplantation; IPI = International Prognostic Index; LDC = low-dose chemotherapy; LDH = lactate dehydrogenase; LVEF = left ventricular ejection fraction; R/R = relapsed-refractory; SPD = sum of the product of the diameters.

A total of twenty-five confounders were identified in the eleven publications. All publications identified the number of prior lines of treatment before CAR-T and diagnosis (disease histology, cell of origin, double or triple hit, NHL subtype, molecular subtype) as potential confounders. In ten publications, prior hematopoietic stem cell transplantation (HSCT), whether autologous or allogeneic, was identified as a potential confounder, along with Eastern Cooperative Oncology Group Performance Status (ECOG PS). Nine publications also identified relapsed/refractory (R/R) status—encompassing factors such as disease status, number of relapses, time to first relapse after diagnosis, refractoriness, time to relapse post-ASCT, and best response (complete remission, CR) to the last therapy—as confounders. The full results are presented in Figure 2.

Figure 2 further highlights a discrepancy between the frequency with which a confounder was identified and how often it was used as an adjustment variable in the ITCs. For example, while ECOG PS was identified as a confounder in ten publications, it was only used for adjustment in seven, primarily due to missing data.

## 4. Discussion

Through our rapid literature review, we identified eleven unanchored ITCs on CAR-T therapies for DLBCL. The potential confounders identified in these studies are widely recognized in clinical practice. The ECOG-PS was mentioned in ten of the studies and is also part of the International Prognostic Index (IPI), a widely used tool for predicting treatment outcomes in DLBCL. The IPI model was identified as a potential confounder in seven ITCs. It includes five independent prognostic factors: age (≤60 or >60 years), Ann Arbor stage (III/IV), elevated LDH (>1× normal), ECOG-PS (≥2), and the number of extranodal sites (>1) [33]. The IPI has been validated and updated, including the revised International Prognostic Index (R-IPI) and the National Comprehensive Cancer Network IPI (NCCN-IPI), which offers better discrimination of high-risk patients [34]. Recent data have demonstrated a clear association between a high-risk IPI score and poorer progression-free survival (PFS) in patients with R/R DLBCL treated with CAR-T therapies. Additionally, the IPI has been linked to overall survival (OS) and neurotoxicity [35]. While the IPI is a composite of ECOG-PS, disease stage, age, LDH, and extranodal sites, each of these factors significantly impacts outcomes individually. For instance, a cohort study of 116 patients with DLBCL in a third-line setting identified risk factors such as ECOG-PS ≥ 2, stage III/IV disease, ≥2 extranodal sites, elevated LDH, increased C-reactive protein (CRP), high IPI at the time of decision (TD) and treatment (TT), and bulky mass [36]. Real-world data have also confirmed these findings, such as the French DESCAR-T registry, which identified high LDH, time to CAR-T failure < 30 days, and elevated CRP at infusion as predictors of OS [37]. According to the SEER platform, an increase in age is associated with a decline in 5-year survival rates, consistent with findings from both real-world data and randomized trials [38,39,40]. While age and disease incidence are known factors, sex appears to have minimal impact on treatment outcomes, despite being included in most ITCs [36].

Diagnosis was another confounder identified in all the ITCs. This encompasses disease histology, cell of origin (COO), double/triple hit gene rearrangements, NHL subtype, and molecular subtype. Gene expression profiling can distinguish between the two main molecular subtypes of DLBCL: the germinal center B-cell-like (GCB) and activated B-cell-like (ABC) subtypes [41]. These subtypes, arising from different COOs, may influence how well patients respond to treatment, with the ABC subtype generally showing poorer outcomes than the GCB subtype [40]. Sehn and Salles (2021) highlighted the clinical relevance of COO subtyping, as targeted therapies may be preferentially effective in one subtype over the other [40].

Bridging therapy (BT), identified in seven ITCs, is an anti-tumor therapy given to patients during the CAR-T manufacturing process. Clinical and real-world data indicate that most CAR-T patients receive BT, often due to higher tumor burden or more rapidly progressing disease, making it a negative prognostic factor. In patients with chemo-refractory disease, conventional BT approaches can be ineffective, and many patients fail to reach CAR-T infusion [42,43,44,45]. A recent study involving 375 patients with large B-cell lymphoma treated with either axi-cel or tisa-cel demonstrated that response to BT significantly increased the likelihood of durable remission, regardless of the bridging modality used, with a marked reduction in the risk of disease progression or death in those with a complete or partial response to BT [43]. Additionally, metabolic tumor volume (TMTV) is a strong prognostic factor in DLBCL and other lymphoma subtypes, though the definition of “bulky disease”, identified in five ITCs, varies among studies (ranging from 5 to 10 cm) [46,47,48,49,50]. While the identified confounders are clinically plausible, the methodology used to select them in the ITCs lacked transparency. None of the studies disclosed their literature search strategy or the expert validation process for the literature review. Consequently, it remains unclear whether all potential confounders were identified and properly analyzed. For instance, the CAR-HEMATOTOX score is a validated prognostic index for patients undergoing CAR-T [51] and was not included in any of the ITCs. Moreover, the eleven publications did not use a consistent set of confounders, highlighting significant heterogeneity in confounder selection. Even commonly considered variables in Health Technology Assessments (HTAs), such as age, sex, and disease severity, were inconsistently included across the ITCs. Notably, only the number of prior treatment lines and diagnosis were consistently identified as potential confounders. However, missing data in some studies prevented their inclusion in matching or sensitivity analyses.

Another issue identified in many publications was the lack of distinction between confounders, prognostic factors, and treatment modifiers, with these terms often being used interchangeably. This had limited impact, however, since all ITCs included in the review were unanchored MAICs, requiring authors to adjust for all identified variables. To improve the reliability and consistency of results, we recommend establishing standardized methodological guidelines for identifying and selecting relevant adjustment variables.

This rapid review has several limitations. First, our search was confined to a single data source (PubMed) and was restricted to full-text articles. Moreover, given the rapidly advancing field of cell and gene therapies—illustrated by the 750 CAR-T therapies in development as of 2022 [52]—ongoing research will be crucial to incorporate emerging data and therapies.

## 5. Conclusions

This rapid literature review identified significant variability and methodological limitations in unanchored ITCs assessing CAR-T therapies for DLBCL. While key clinical confounders like the IPI and diagnosis were commonly recognized, inconsistent selection and reporting across studies undermine comparability. Standardized guidelines are needed to ensure consistent identification of confounders and improve the reliability of future analyses.

## Figures and Tables

**Table 1:** Keywords used in PubMed.

Technique	Indirect comparison*, treatment comparison*, simulated treatment comparison*, STC, network meta analys*, NMA, MAIC, matching adjusted indirect comparison*, adjusted comparison*, comparing efficacy, real world comparison*, comparative efficacy
Drug	chimeric antigen receptor T-cell therap*, CAR T*, tisagenlecleucel, tisa-cel, Kymriah, axicabtagene ciloleucel, axi-cel, Yescarta, lisocabtagene maraleucel, liso-cel, Breyanzi
Indication	diffuse large B cell lymphoma, large B cell lymphoma, DLBCL, LBCL

**Table 2:** Literature review capturing disclosed methodology of publications on indirect comparisons of CAR-T in DLBCL.

Comparison	Confounders	Endpoints	Method	Publication
Axi-cel vs. tisa-cel (third line)	Age Sex LDH CRP ECOG PS Diagnosis Bulk assessed at lymphodepletion Ann Arbor stage Treatment center Bridging and response to bridging Prior HSCT Number of prior lines of treatment before CAR-T Time between last treatment and infusion Time between first CAR T order and infusion	OS and PFS for all confounders	Univariate prognostic analyses Type of ITC: Propensity score matching (PSM) Inverse probability of treatment weighting was used to support the findings of PSM analysis and to allow for proper comparison between the two populations	Bachy, et al. [20]
Liso-cel vs. tisa-cel (third line)	Clinical factors included in the primary and sensitivity analyses: ECOG PS (all endpoints) Active secondary CNS lymphoma (all endpoints) Disease histology (all endpoints) Prior allo-HSCT (all endpoints) Prior auto-HSCT (all endpoints, except PFS for primary analysis) R/R to last therapy (all endpoints, except OS for primary analysis) CrCl pre-lymphodepletion (all endpoints except OS and CRR for primary analysis) Other variables not included in the primary MAIC but included in the sensitivity analysis: 8. Age (PFS and OS) 9. Number of prior therapies (all endpoints) 10. Sex (PFS and OS) 11. IPI (all endpoints) 12. Stage of disease (all endpoints) 13. Cell of origin (all endpoints) 14. Double or triple hit (all endpoints) 15. LVEF at screening (all endpoints) 16. ALC pre-leukapheresis (all endpoints) NB: bridging therapy, while identified as a clinical factor at baseline, was not included in the MAIC (primary or sensitivity analysis), because both trials allowed its use per the investigator’s discretion.	CR, ORR, OS, and PFS	Literature search reviewed by a panel of external clinical experts (ranked list using classification or survival-based random forest models) Type of ITC: unanchored MAIC + propensity score matching	Cartron, et al. [21]
Liso-cel vs. axi-cel (third line)	Clinical factors included in the primary and sensitivity analyses: Disease histology (all efficacy endpoints) ECOG PS (all efficacy and safety endpoints) Secondary CNS lymphoma (all efficacy and safety endpoints) Prior allo-HSCT (all efficacy and safety endpoints) Tumor burden (all efficacy and safety endpoints) IPI score (PFS and OS; CRR for sensitivity analysis only) R/R to last therapy (all efficacy endpoints) Bulky disease (CRR, PFS, OS; ORR for sensitivity analysis only) Age (OS; PFS, CRR, ORR for sensitivity analyses only; for safety endpoints) Prior auto-HSCT (ORR and CRR; PFS and OS for sensitivity analyses only; for safety endpoints) Disease stage (ORR; CRR, PFS, OS for sensitivity analyses only) CrCl (ORR and PFS; CRR and OS for sensitivity analyses only) Extranodal disease (CRR; ORR, PFS, OS for sensitivity analyses only) Other variables used for sensitivity analysis: 14. Number of prior lines of therapy (all efficacy and safety endpoints) 15. Sex (all efficacy endpoints) 16. LVEF (all efficacy endpoints) 17. ALC (all efficacy endpoints) NB: bridging therapy use was only selected for initial matching analysis. The reason for not including bridging therapy in the sensitivity analysis was to assess its effect on outcomes.	Efficacy: ORR, CRR, PFS, and OSSafety: all grade and grade ≥ 3 CRS, neurological events, grade ≥ 3 infections, hypogammaglobulinemia, grade ≥ 3 prolonged cytopenia	Literature search and input from a panel of five external clinical experts (Canada, France, Germany, UK, US) (ranked list using statistical random forest models) Type of ITC: unanchored MAIC + Propensity score weighting	Maloney, et al. [22]
Tisa-cel vs. SOC (salvage therapy, CORAL cohort) (third line)	Confounders identified as very important and included in the matching analysis: Age at initial diagnosis Ann Arbor stage disease Extranodal site involvement Status of disease (R/R status) Time to first relapse after diagnosis Prior HCT Number of relapses Confounders identified as very important but not included in the analysis due to missing data: 8. Serum LDH level 9. ECOG PS 10. Double/triple gene hit 11. Bulky disease Other confounders identified as less or not important and not included: 12. Molecular subtype (less important) 13. Hepatitis B infection (not important) Other baseline variables: Age Sex Ann Arbor disease stage at diagnosis IPI Number of prior lines of therapies	OS and ORR for all confounders	Systemic literature search and clinical experts’ inputs (ranking with “not important”, “less important”, and “very important”) Type of ITC: Propensity score weighting based on both standardized mortality ratio weight (SMRW) and fine stratification weight (FSW)	Maziarz, et al. [23]
Axi-cel vs. tisa-cel (third line)	IPI score ECOG PS Disease stage Refractoriness (relapsed/refractory) Double/triple hit Cell of origin Number of prior therapies NB: use of bridging therapy was identified as a covariate but not used in MAIC because of data availability (no bridging chemo in axi-cel’s trial)	ORR, CR, and OS for all covariates	Inputs from clinical experts (identification and ranking) Type of ITC: unanchored MAIC + propensity score weighting	Oluwole, et al. [24]
Liso-cel vs. SOC (salvage therapy) (third line)	NHL subtype Sex Age Prior ASCT R/R status to last therapy Disease stage IPI score NB: ECOG PS, LDH levels, and prior lines of therapy are not part of the MAIC due to missing data	OS, CRR, and ORR for all covariates	Baseline characteristics reported in TRANSCEND and SCHOLAR-1 Type of ITC: unanchored MAIC + propensity score weighting	Salles, et al. [25]
Tisa-cel vs. liso-cel(3rd line)	Age Sex Disease histology ECOG PS LVEF SPD LDH Prior HSCT Number of prior lines of therapy, Received bridging chemotherapy Refractory status to prior therapies NB: fludarabine-based LDC was included for sensitivity analysis	ORR, CRR, OS, and PFS for all confounders	Input from clinical experts (identification and validation) and factors considered important by Abramson, et al. [26] Type of ITC: MAIC + propensity score weighting	Schuster, et al. [27]
Axi-cel vs. pooled experimental CAR-Ts (third line)	Age Disease stage Disease histology Refractory status Number of prior lines of therapy Extranodal disease status NB: IPI score and ECOG PS, while important covariates, are not part of MAIC due to missing data in experimental CAR-Ts trials	Efficacy: PFS for all covariates Safety: grade ≥3 CRS and neurotoxicity for all covariates	Selection of covariates following the NICE Guidelines [10] and confirmed by clinicians and experts Type of ITC:unanchored MAIC	Weinstein, et al. [28]
Tisa-cel vs. axi-cel (third line)	Predominant disease histology IPI ECOG PS Number of prior lines of therapy History of refractory Double/triple hits Sex Prior ASCT Time to relapse post-ASCT Bulky disease Bridging chemotherapy (only in the prediction model, not the MAIC) Fludarabine-based LDC (only in the prediction model, not the MAIC) Bendamustine-based LDC (only in the prediction model, not the MAIC)	OS for all endpoints	Literature research (Oluwole, et al. [29]) and patient-level data from JULIET trial Type of ITC: MAIC + propensity score matching, and simulated treatment comparison (STC)	Zhang, et al. [30]
Tisa-cel vs. salvage chemo (third line)	Key prognostic factors include: Number of prior relapses Prior SCT	OS	No methodology disclosed Type of ITC: MAIC	Moradi-Lakeh, et al. [31]
Axi-cel vs. Liso-cel (third line)	Effect modifiers included in the MAIC: ECOG PS (0 vs. ≥1) Best response of CR to last therapy Use of bridging therapy LBCL subtype (DLBCL vs. HGBCL vs. TFL vs. others) Number of prior lines of systemic therapy (1 vs. 2 vs. 3 vs. ≥4) Prior auto-SCT Tumor burden by the sum of product diameter (SPD; <50 vs. ≥50 cm^2^) Age (<65 vs. ≥65 years) LDH (<500 vs. ≥500 U/L) NB: use of bridging therapy was identified as a covariate but not used in MAIC because of data availability (no bridging chemo in axi-cel’s trial); therefore, it was only adjusted within the scenario analysis Other variables not included in the primary MAIC but included in the post hoc sensitivity analysis: IPI Disease stage Bulky disease Extranodal disease	Efficacy: DoR, PFS, and OS for all effect modifiers Safety: CRS, neurological events for all effect modifiers	Effect modifiers were ranked according to clinical relevance based on recommendations from clinical experts and data availability Type of ITC: MAIC + propensity score weighting	Oluwole, et al. [32]

Legend: ALC = pre-leukapheresis absolute lymphocyte count; allo-HSCT = allogeneic hematopoietic stem cell transplantation; auto-HSCT = autologous hematopoietic stem cell transplantation; bendamustine-based LDC = bendamustine-based low-dose chemotherapy; CNS = central nervous system; CrCl = creatinine clearance; CRP = C-reactive protein; CRR = complete response rate; CRS = cytokine release syndrome; DoR: duration of response; ECOG PS = Eastern Cooperative Oncology Group Performance status; fludarabine-based LDC = fludarabine-based low-dose chemotherapy; IPI = International Prognostic Index; LDH = lactate dehydrogenase; LVEF = left ventricular ejection fraction; MAIC = matching-adjusted indirect comparison; NICE = National Institute for Health and Care Excellence; NHL = non-Hodgkin‘s lymphoma; ORR = overall response rate; OS = overall survival; PFS = progression-free survival; R/R = relapse/refractory; SPD = sum of the product of the diameters.

## Supplementary Materials

The following supporting information can be downloaded at: https://www.mdpi.com/article/10.3390/cancers17081335/s1, PRISMA 2020 Checklist.
